# Sensory processing sensitivity and social pain: a hypothesis and theory

**DOI:** 10.3389/fnhum.2023.1135440

**Published:** 2023-06-14

**Authors:** Lucia Morellini, Alessia Izzo, Alessia Celeghin, Sara Palermo, Rosalba Morese

**Affiliations:** ^1^Faculty of Biomedical Sciences, Università della Svizzera italiana, Lugano, Switzerland; ^2^Department of Psychology, University of Turin, Turin, Italy; ^3^Neuroradiology Unit, Diagnostic and Technology Department, Fondazione Istituto di Ricovero e Cura a Carattere Scientifico (IRCCS), Istituto Neurologico Carlo Besta, Milan, Italy; ^4^Faculty of Communication, Culture and Society, Università della Svizzera italiana, Lugano, Switzerland

**Keywords:** sensory-processing sensitivity, highly sensitive people, pain, social pain, Cyberball Game

## Abstract

Sensory-processing sensitivity (SPS) defined, as a personality trait, seems to be characterized by emotional sensitivity, and stronger reactivity to both external and internal stimuli. SPS can represent a risk factor for developing clinical conditions during childhood and adolescence. This personality trait is not to be considered a pathological clinical condition, however, can expose to greater environmental vulnerability. In particular, the recent studies about SPS can be contextualized to social situations that evoke traumatic and stressful emotional responses such as social exclusion. We hypothesize that highly sensitive people (HSP) are more vulnerable to social exclusion and social pain. This hypothesis could help structure new educational and intervention models designed to improve coping strategies and promote HSP’s psychophysical and social well-being.

## Introduction

### Highly sensitive people: conceptualization and assessment methods

Highly sensitive people (HSP) were first studied and described by psychologist Aron (1996), who identified people that are high in a personality trait known as sensory-processing sensitivity (SPS). Individuals with high sensitivity exhibit abnormal reactivity and emotional sensitivity to both external and internal stimuli which, in turn, might interfere with daily life. High SPS is seen in 20% of the populations studied (Aron and Aron, 1997); recently Lionetti et al. (2019) indicate around 30% of the people (Lionetti et al., 2018, 2019; Pluess et al., 2018). It seems equally distributed in both genders (Kagan and Kagan, 1994; Kristal, 2005). Importantly, SPS was identified as a one-dimensional construct, differing from introversion, emotionality, neuroticism, and shyness. SPS seems only related to high sensitivity (Aron and Aron, 1997).

The sensitivity trait represents the result of a careful strategy of processing environmental information, carried out before taking an action (Aron et al., 2012). This strategy can lead to two outcomes - more adaptive or less adaptive depending on the circumstances: increased awareness of environmental details or overstimulation resulting in anxiety or avoidance conduct (Aron, 2018). Research on this topic states that deeper processing (i.e., a more meaningful analysis of information) of stimuli would not be stimulus-specific but is the result of high responsivity to salient environmental stimuli (Aron et al., 2012). The evolutionary purpose of this kind of information processing is to utilize information obtained in situation A (where more details are noticed) to better predict the future and enact more effective responses in situation B (the same as situation A, where people practise the behaviors previously learned) (Aron et al., 2012; Aron, 2018).

It is important to underline that SPS is not to be considered a pathological clinical condition, however high sensitivity could expose to an increased risk of developing psychopathologies, especially during childhood and adolescence (Aron, 2018; Greven et al., 2019; Lionetti et al., 2022). In particular, High SPS children who have had an unhappy childhood or have been exposed to harmful environments would be more vulnerable to anxiety, depression, and high levels of perceived stress with physical issues such as pain or fainting (Liss et al., 2005; Benham, 2006). In contrast, children who had a happy childhood and grew up in a supportive environment would develop several adaptive advantages, even to a greater extent than non-sensitive children (Pluess and Belsky, 2013).

In such a case, it is possible to detect advantageous sensitivity, that is the propensity to disproportionately benefit from positive experiences and contexts (Pluess and Belsky, 2013, 2015). Conversely, individuals made vulnerable may have a greater propensity to succumb to the negative effects of adversity, as explained by the diathesis-stress model. The two evolutionary directions are not mutually exclusive (Belsky and Pluess, 2009; Bakermans-Kranenburg and van Ijzendoorn, 2011). In fact, the SPS would likely be a predictor of an increased likelihood of benefiting from positive contexts and situations although it exposes a person to greater potential vulnerability (Belsky, 2005; Pluess and Belsky, 2009).

A recent study explored the *vantage sensitivity hypothesis* to understand possible individual differences in response to psychotherapist treatment (de Villiers et al., 2018). According to the Authors, individual differences in response to environmental exposure can be explained by the diathesis–stress model, which conceives psychological vulnerability as due to the interaction between an individual’s inherent propensity for vulnerability, and some sort of external life stressor. Endogenous markers of vantage sensitivity appear to fall into *genetics* (5-HTTLPR); *physiology* (cortisol reactivity), and *psychological traits* (childhood negative emotionality) with several factors of vulnerability (Obradović et al., 2010; Ramchandani et al., 2010; Drury et al., 2012; Pluess and Belsky, 2015; Bozzatello et al., 2019, 2021; Longobardi et al., 2020; Morese and Longobardi, 2020). Several higher-order cognitive processes involve individual sensitivity markers and represent potential facilitators of vantage sensitivity (or environmental sensitivity more generally): attentional processes, reward sensitivity, social sensitivity, and stress reactivity (Drury et al., 2012).

Importantly, the concept of *environmental sensitivity* can encompass differential susceptibility, vantage sensitivity, biological sensitivity to context, and *SPS* at the same time (de Villiers et al., 2018). The centrality of the nervous system in that context can be evaluated in terms of “neurosensitivity,” which in turn would manifest itself physiologically and at the psychological level. In addition, the quantifiable SPS neurobiological trait influences the degree of sensory stimuli processing (de Villiers et al., 2018). Authors through the review of literature indicate interesting results about a growing number of studies that report that some people with heightened sensitivity benefit more from psychological intervention than others.

The implication of the above is particularly important: it means that environmental sensitivity factors explain individual differences in response to both adverse and supportive experiences, such as psychotherapy. Therefore, knowing individual sensitivity could help in the strategy and the design of clinical practice according to a tailored patient-centered approach.

The topic has been studied empirically, but the ability to predict clinical outcomes remains limited (Pluess and Boniwell, 2015; Nocentini et al., 2018). It has been reported, however, that targeting interventions can have a number of practical advantages, both financially and in terms of improving clinical services. Indeed, individuals with higher levels of sensitivity and responsiveness may require shorter interventions, while those with higher levels of resilience may require longer, more intense interventions or different strategies. According to de Villiers et al. (2018), this assumption can also be applied to educational and social welfare plans.

On the other side, SPS may expose highly sensitive people to an increased risk of psychopathology. Indeed, Individuals with SPS report, for example, increased frequency and distress of nightmares (Carr et al., 2021). SPS has been previously found in autism spectrum disorder (Joosten and Bundy, 2010), attention deficit hyperactivity disorder (Panagiotidi et al., 2020), mild cognitive impairment, and dementia (Rhodus et al., 2022).

### Assessment approach: diagnostic factors and proposed rating scales

In 2012, Aron published a meta-analysis in which she analyzed 4 typical features of HSP: inhibition of behavior, greater sensory awareness, deep information processing, and stronger and more emphasized emotional responses. These are summarized and conceptualized in the acronym DOES (Aron, 2018):

• *Depth of Processing*: deep information processing usually involves elaboration rehearsal, requiring a more meaningful analysis of information and leading to better recall. HSP exposes each stimulus to more processing, making comparisons and/or connections with past, similar situations, or other objects. Processing is slower and more painstaking and can be either conscious or unconscious. In HSP brain areas devoted to deep information processing have been found to be more elicited (Jagiellowicz et al., 2011). The area most activated is the insula, a core area for awareness of emotional and inner states, and salient stimuli (Acevedo et al., 2014).
• *Overarousability*: excessive attention to environmental details in HSPs leads to overstimulation and early and high fatigue (Gerstenberg, 2012). HSPs are usually faster and more accurate, but at the same time, more stressed and exhausted than non-sensitive people, especially in impulsive and risk-taking behaviors (Gerstenberg, 2012).
• *Emotional responsiveness/empathy* (emotional reactivity): HSPs are more reactive than non-HSPs when exposed to images with positive valence (Jagiellowicz et al., 2011). This phenomenon is reinforced according to the emotional valence experienced during childhood (Pluess and Belsky, 2013). HSPs are also more empathic and have better skills in mentalization and Theory of Mind (Acevedo et al., 2014).
• *Sensitivity to subtle stimuli and details*: HSPs can notice small details that escape others. This is a matter of complex and careful sensory processing (Aron, 2018).

Considering the above, a reliable assessment approach to HSP is the use of standardized questionnaires such as:

• The *Highly Sensitive Person Scale* (HSP; Aron and Aron, 1997) with 27-item is a self-report to detect environmental sensitivity in adults. Scores > 14 indicate the possible presence of HSP traits.
• The *Highly Sensitive Person Scale – Brief Version* (HSP-12; Pluess et al., 2023) is a self-report that uses only 12-items to assess Environmental Sensitivity in adults.
• The *Highly Sensitive Child Scale* (HSC; Pluess et al., 2018) scale is self-report with 12-item designed for children and adolescents (between 8 and 18 years) to evaluate Environmental Sensitivity.

In addition to the DOES (Aron, 2018), other typical characteristics of a highly sensitive individual are considered in these rating scales. Examples are conscientiousness, creativity, spirituality, and love of nature, but also, higher physical sensitivity, a more responsive immune system, and higher pain sensitivity (Table 1).

**TABLE 1:** List of distinguishing characteristics of highly sensitive persons considered childhood adulthood comparison.

HSP in childhood	HSP in adulthood
● The child tends to get particularly anxious about new situations, more so than peers, gasps easily, does not cope well with big changes, and generally does not appreciate big surprises	● Preference in remaining on the edge of a situation for a moment before entering it; exploration occurs first through observation and reflection
● The child prefers quiet, often solitary, and sometimes repetitive games (“favorite games or books”), considers whether it is safe before playing “dangerous” games	● Great awareness of details and minute changes
● The child tends to show interest and attention to others, especially considering vulnerable children and their emotions	● Consider every possible consequence before acting: “do it once and do it best.”
● The child reacts strongly to what he perceives as injustices, to himself but sometimes to others as well	● Perception of others’ emotions and feelings, intuited from non-verbal details
● The child is a perfectionist, has high expectations of his/her own performance, gets easily upset if he/she fails at something or loses in a game	● Great capacity for empathy and emotional connection
● On such occasions, the child may exhibit much more explosive and angrier behavior than usual and broods long after possible failures and it is difficult to calm him down	● Greater impact of the surrounding emotional environment, both negatively and positively, in childhood history and adulthood
● The child broods over any form of criticism or teasing he/she receives, especially from peers, and particularly suffers from it, struggling to “let it slide off”	● Particularly conscientious acting with great attention to the link between causes and consequences
● The child learns better from gentle correction than strong punishment, and generally struggles to share rules and punishments if he/she does not fully understand their meaning.	● Particular intolerance and bewilderment with respect to injustice, environmental concerns, compassion
● The child seems very intuitive, to the point of sometimes seeming to be able to read minds	● Frequent overstimulation, overload from overactivation (worse performance) but also from hypoactivation (boredom): necessary balance
● The child is very attentive to even the slightest changes in the environment, notices even the smallest details, especially social ones, and easily perceives and absorbs family tensions	● Talent, passion, or attraction with respect to art forms
● It may happen that the child acts as a mediator or restrains his/her emotions so as not to make the situation worse	● Interest in less material, and therefore deeper and more spiritual aspects
● The child can empathize with the moods of others, even non-family members and is particularly attentive to the needs of others, and easily notices their distress.	● Great emotional reactivity to events, subjectively more intense than others
● The child is not prone to teasing or insulting other children; he/she can be protective at times	● Particular stress with respect to change
● The child tends to follow general rules conscientiously, almost too rigidly	● Dreams are often vivid and full of detail
● At times, the child appears visibly older than his/her age because of his/her ability to intuit even what is unspoken; he/she is very curious to go deeper, even on complex topics, and can be surprising in the ability to associate with situations and concepts, even if no one has ever explained them before	● Recognition of such characteristics since childhood
● The child feels things deeply and often asks deep, reasoned and provocative questions.	● Overstimulation in environments with bright lights or loud noises (this may not manifest in adolescence)
● The child takes parental promises very literally and learns quickly whether these premises are fulfilled or not, and in the latter case manifests much annoyance	● Frequent physical reactions, more reactive immune response, particular sensitivity to pain, stimulants, drugs
● The child loves contact with art, music, fantasy and nature, especially animals	● Often indirect mode of communication, considering the possible reaction in the interlocutor
● The child is disturbed by noisy places, dislikes chaotic situations and social situations in which he/she has to deal with unfamiliar environments and people, or face new tests and tasks in which he will be judged	● Deep contact with nature, with a calming effect, fondness for animals, plants, and pleasure in being in or near water
● When subjected to situations of overstimulation the child may suddenly exhibit very oppositional and defiant behavior, even to the point of becoming aggressive and uncaring of others; he/she also has difficulty falling asleep after a particularly stimulating/exciting day	
● The child is particularly sensitive to pain, and tends to somatise discomforts and fears, for example in headaches or stomach aches and a general lowering of the immune system	
● The child protests against tight or rustling clothes, shoe seams or clothing labels, feels the urgency to change clothes e.g., when they are wet or silted up	

### Current evidence: HSP, physical and social pain

Sensory-processing sensitivity represents a trait that highlights interindividual differences in sensitivity to environments that can encompass a wide range of internal and external stimuli, either conditioned or unconditioned, as well as physical environments, sensory environments, social environments, and internal events (such as thoughts, feelings, and bodily sensations for example for hunger and pain) (Greven et al., 2019).

The anterior cingulate cortex (ACC), the anterior insula (AI), and the primary and secondary somatosensory cortex (S1 and S1) have been considered as hubs of physical pain (Eisenberger, 2012). More recently, neuroimaging and neurophysiological evidence reported that noxious stimuli activated neural correlates and connectivity in several cerebral areas: insular cortex, prefrontal cortex, somatosensory cortex, ACC, thalamus (Mercer Lindsay et al., 2021).

Research on the neural correlates of sensitivity to physical pain associates it with cortical thickening in S1, posterior cingulate cortex, and the orbitofrontal cortex (Hsiao et al., 2020). Nonetheless, (hyper)activation of some of these regions in pain-sensitive individuals than in pain-insensitive individuals has been also observed (Coghill et al., 2003; Erpelding et al., 2012).

The lingering effects of this pain sensitivity may exacerbate the effects of brain trophism resulting from chronic pain. Chronic pain causes both central and pathology-specific gray matter alterations in several cerebral networks (Cauda et al., 2014). Common alterations have been detected in the prefrontal regions, post and precentral gyri, thalamus, cingulate cortex, anterior insula, basal ganglia, periaqueductal gray, and inferior parietal lobule. As a highly salient stimulus, pain constantly taxes the salience and attentional processing systems, interfering with cognitive abilities and consuming emotional and cognitive resources. Most available studies report an overlapping contribution of salience and attention networks in subjects with various chronic pain conditions and pathologies (Cauda et al., 2014, Palermo, 2022).

Most of the cerebral responses are activated when physical pain is present, but they can also be elicited when pain is not triggered by a physical stimulus (Mouraux and Iannetti, 2018). For example, similar neural underpinnings can be observed in social pain (Eisenberger, 2012). While physical pain is related to actual or potential tissue damage, social pain is associated with psychological distance from other people or social groups (Xiao et al., 2020). Indeed, social pain is considered a strong experience of discomfort and distress caused by ostracism (i.e., social exclusion or loss such as rejection from the others or the own group) that involve affective, behavioral and cognitive iatrogenic consequences (Eisenberger and Lieberman, 2004; Eisenberger, 2012; Wesselmann et al., 2012; Sturgeon and Zautra, 2016; McIver et al., 2019; Kiefer et al., 2021; Schwarz et al., 2021; Jauch et al., 2022). Physical and social pain share not only physiological-adaptive characteristics to the environment, psychological and behavioral manifestation, but also a strong capacity for interaction and interpenetration (Xiao et al., 2020). Nevertheless, social pain neurobiology has been studied for much less time.

The “Cyberball Game,” a virtual task in which participants pass the ball with two virtual players, has been used in neuroimaging studies to understand the neurophysiological basis of social pain (or ostracism). At first, both players pass the ball normally, but after a while they begin passing it only to one another, excluding the real player (Hartgerink et al., 2015). The consequences of exclusion from the game evoke negative feelings of discomfort and unpleasantness and activate neural areas devoted to processing negative affect, and shared with neural substrates of physical pain: the dorsal ACC, the subgenual ACC, and the anterior insula (AI) (Coghill et al., 2003; Masten et al., 2009, 2011; Eisenberger, 2012; Erpelding et al., 2012; Cacioppo et al., 2013; Novembre et al., 2015; Rotge et al., 2015; Auriemma et al., 2020; Hsiao et al., 2020).

The possible overlap of physical pain with social pain leads us to hypothesize that highly sensitive individuals are hyper-reactive to both types of pain, but to date there is no evidence on the relationship between pain sensitivity and high HSP scores.

A recent study on ostracism was concerned with understanding how behavioral and neural correlates could be modulated by social support (Morese et al., 2019). The authors administered the Cyberball Game to their normal sample, as modified by Novembre et al. (2015). Through functional Magnetic Resonance Imaging (fMRI), participants were observed to activate their brains in response to receiving the emotional, appraisal, or no social support from a friend (Lo Gerfo et al., 2019; Morese et al., 2019). At the neural level, social-emotional support (hand caresses) reduced feelings of unpleasantness and reduced AI recruitment, a core brain area associated with negative emotional states both in social pain and in physical pain (Morese et al., 2019; Palermo, 2022). In contrast, social appraisal support (text messages) would not attenuate the effects, but on the contrary, would increase feelings of unpleasantness (Morese et al., 2019). On a neural level, the right temporal parietal junction (TPJ) is less activated (a component of the ToM network that detects inconsistencies and distinguishes oneself from others), resulting in a reduced need to comprehend the social situation (Lo Gerfo et al., 2019; Morese et al., 2019). In line with this finding, an increase in subACC activity (i.e., an increase in negative affectivity) was also observed (Morese et al., 2019).

Although there are no relevant studies on this yet, we can speculate, given the wide range of evidence (both neural and behavioral) in support, that a highly sensitive individual is hyper-reactive not only to physical pain but also to social pain. Consequently, social-emotional support and not just social appraisal support might prove to be an effective supplement to traditional psychotherapies.

In support of this hypothesis, a recent study conducted by Ren et al. (2020) suggested a possible relationship between pain sensitivity and empathy. Authors found that individuals with high pain sensitivity would experience higher empathy for other people’s pain, manifesting it through stronger emotional reactions. Importantly, a significant correlation between *pain sensitivity* and *emotional empathy* in the context of a mediation-moderation induced by anxiety, catastrophizing, and fear was found. Those behavioral findings are consistent with meta-analytic connectivity analysis on experimental pain (Palermo et al., 2015). A highly distributed perceptual set of self-regulation can prompt brain areas to elaborate information in which emotion, action, and perception represents an important role (Palermo et al., 2015). Highly sensitive metacognitive-executive functions (such as ToM and mentalizing) and the intensity of emotional reactions normally associated with highly pain-sensitive individuals can therefore be considered prototypical characteristics of HSPs. What has been described leads us to speculate that social pain resulting from the SPS trait may be significantly perceived as greater in HPSs.

### Hypotheses

Understanding how hypersensitivity, negative situations, and expectations influence nociception is important to understanding how nocebo responses are neuromodulated in HSPs. The implications that result are of great clinical-applicative importance since new approaches for the management of physical and social pain in these people are needed.

Nocebo hyperalgesia could represent the outcome of negative anticipations leading to extended pain experiences (Tracey, 2010). Acute psychological stress affects nocebo responses and, consequently, the pain threshold, through its action on serotonin, dopamine, and norepinephrine (Palermo, 2022). Because dopamine and norepinephrine specifically potentiate the ability to process and modulate pain, it is critical that these neurotransmitters are well-balanced (Palermo, 2022).

Highly sensitive people are more prone to stress (Gulla and Golonka, 2021). The reason could be attributed to genetic mutations found in HSPs in the three neurotransmitters directly related to stress and pain tolerance (Chen et al., 2011). In a stressed HSP, neurochemicals are dysregulated, deteriorating the nervous system’s ability to cope with both short-term and long-term pain (Chen et al., 2011).

Anxiety, stress, a sense of overload, and catastrophism impact on general well-being (Gulla and Golonka, 2021) and pain expectancy (Palermo et al., 2015) and trigger hyperalgesia phenomena (nocebo effects), easily verified in psychobiological responses associated with pain anticipation (Palermo et al., 2015; Amanzio and Palermo, 2019). Therefore, the psychological mechanisms of emotion management become fundamental. Indeed, modulatory cortical networks involved in placebo analgesia largely overlap with those involved in the regulation of emotional processes, while nociceptive brain networks are downregulated in parallel with behavioral analgesia (Amanzio et al., 2013).

According to Gulla and Golonka (2021), attention awareness can moderate the relationship between HSPs and resilience to psychophysical stress, including pain. Indeed, the positive relationship between sensing subtle emotions and tolerating negative emotions is strengthened by attentional awareness. That finding is in line with a conceptualization of executive-metacognitive functions as predictors of disease prognosis and treatment compliance (Morese et al., 2018).

## Conclusion

Highly sensitive people are those who perceive internal and external stimuli more intensely and deeply. This occurs because the trait of High Sensitivity, which is proposed to be partly innate (Aron, 2004) and partly genetic (Aron et al., 2012; Acevedo et al., 2014; Assary et al., 2021), is determined by a different functioning of the neurological system, which is more active and susceptible. In our hypotheses, high SPS decreases well-being in situations of social pain. Proper attention awareness and executive-metacognitive functions may thus be of great importance in improving well-being and protecting HSP from various stressors and, consequently, from the relapses of hyperalgesia or (chronic) physical and social pain.

What is presented here lays the foundation for future studies on HSP and social pain, with use of advanced neuroimaging techniques and exploration of determinants through the use of the proposed rating scales for HSP.

## Data availability statement

The original contributions presented in this study are included in the article/supplementary material, further inquiries can be directed to the corresponding authors.

## Author contributions

LM and AI conducted the literature search and wrote the first draft of the manuscript. AC participated in writing. SP and RM conceived the content of the manuscript, supervised the writing, and wrote the last version of the manuscript. All authors contributed to the article and approved the submitted version.
